# Supplementary material for: Complex‐centric proteome profiling by SEC‐SWATH‐MS
Source: Mol Syst Biol. 2019 Jan 14;15(1):e8438. doi: 10.15252/msb.20188438 (PMC6346213; doi:10.15252/msb.20188438)
Supplement: Supplementary file 8 — Dataset EV7 [file MSB-15-e8438-s008.zip › feature_plots_string/P05198.pdf]

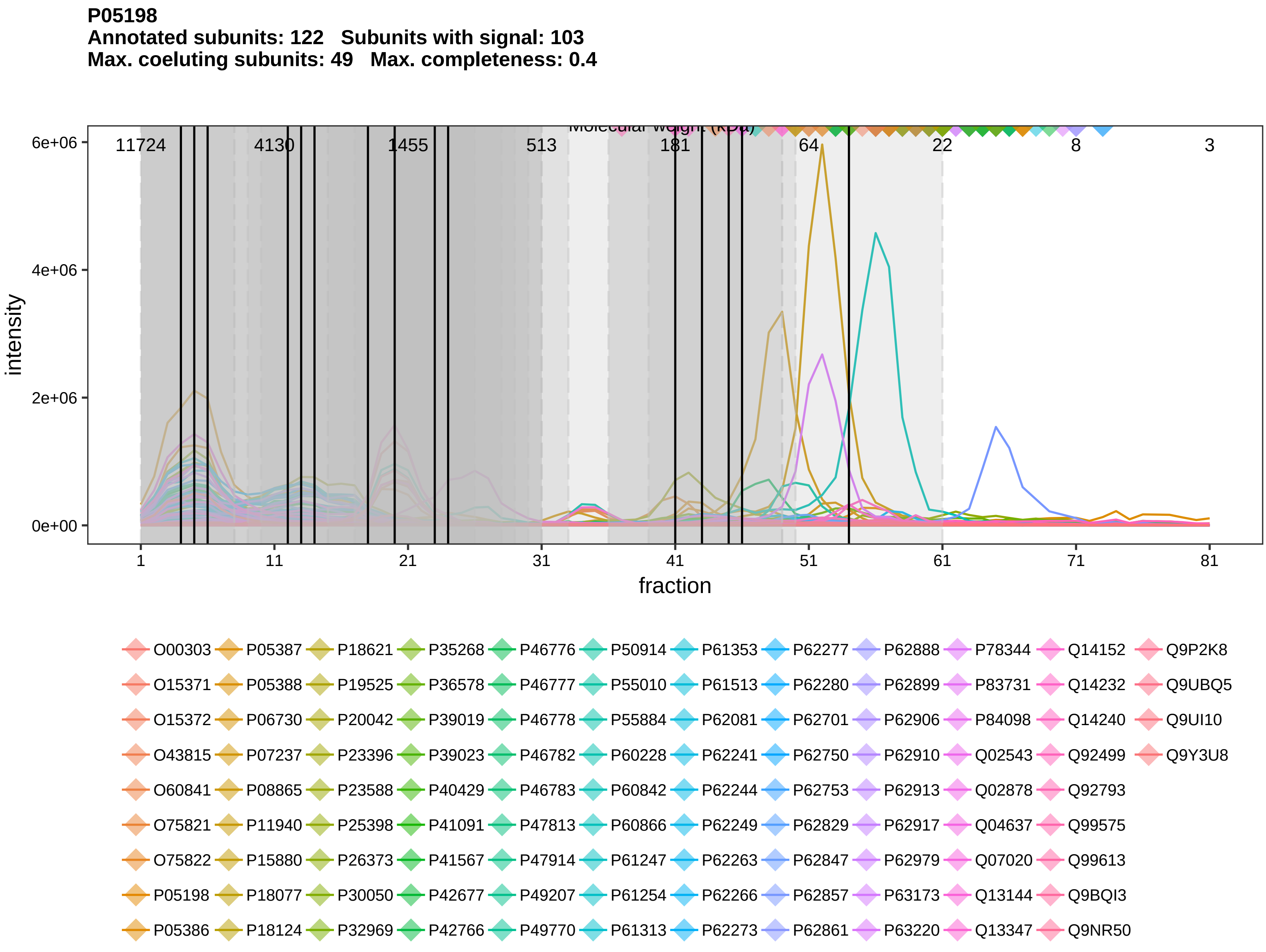
P05198
Annotated subunits: 122 Subunits with signal: 103
Max. coeluting subunits: 49 Max. completeness: 0.4
Molecular weight (kDa)
11724
4130
1455
513
181
64
22
8
3
intensity
6e+06
4e+06
2e+06
0e+00
fraction
1
11
21
31
41
51
61
71
81
O00303
O15371
O15372
O43815
O60841
O75821
O75822
P05198
P05386
P05387
P05388
P06730
P07237
P08865
P11940
P15880
P18077
P18124
P18621
P19525
P20042
P23396
P23588
P25398
P26373
P30050
P32969
P35268
P36578
P39019
P39023
P40429
P41091
P41567
P42677
P42766
P46776
P46777
P46778
P46782
P46783
P47813
P47914
P49207
P49770
P50914
P55010
P55884
P60228
P60842
P60866
P61247
P61254
P61313
P61353
P61513
P62081
P62241
P62244
P62249
P62263
P62266
P62273
P62277
P62280
P62701
P62750
P62753
P62829
P62847
P62857
P62861
P62888
P62899
P62906
P62910
P62913
P62917
P62979
P63173
P63220
P78344
P83731
P84098
Q02543
Q02878
Q04637
Q07020
Q13144
Q13347
Q14152
Q14232
Q14240
Q92499
Q92793
Q99575
Q99613
Q9BQI3
Q9NR50
Q9P2K8
Q9UBQ5
Q9UI10
Q9Y3U8
